# Comparable Postprandial Amino Acid and Gastrointestinal Hormone Responses to Beef Steak Cooked Using Different Methods: A Randomised Crossover Trial

**DOI:** 10.3390/nu12020380

**Published:** 2020-01-31

**Authors:** Utpal K. Prodhan, Shikha Pundir, Vic S.-C. Chiang, Amber M. Milan, Matthew P. G. Barnett, Greg C. Smith, James F. Markworth, Scott O. Knowles, David Cameron-Smith

**Affiliations:** 1 Liggins Institute, The University of Auckland, 85 Park Road, Grafton, Private Bag 92019, Auckland 1023, New Zealand; 2 Department of Food Technology and Nutritional Science, Mawlana Bhashani Science and Technology University, Tangail 1902, Bangladesh; 3 Riddet Institute, Palmerston North 4442, New Zealand; 4 Food Nutrition & Health Team, AgResearch Limited, Private Bag 11008, Palmerston North 4442, New Zealand; 5 The High-Value Nutrition National Science Challenge, Auckland 1023, New Zealand; 6 Department of Pharmacology, Faculty of Medicine, University of New South Wales, NSW 2052, Australia; 7 Department of Molecular and Integrative Physiology, University of Michigan, MI 48109, USA; 8 Food & Bio-Based Products Group, AgResearch Limited, Private Bag 11008, Palmerston North 4442, New Zealand; 9 Singapore Institute for Clinical Sciences, Agency for Science, Technology, and Research, Singapore 117609, Singapore

**Keywords:** beef, protein digestion, amino acids, sous-vide cooking, pan-frying

## Abstract

Cooking changes the texture and tenderness of red meat, which may influence its digestibility, circulatory amino acids (AA) and gastrointestinal (GI) hormonal responses in consumers. In a randomised crossover intervention, healthy males (*n* = 12) consumed a beef steak sandwich, in which the beef was cooked by either a pan-fried (PF) or sous-vide (SV) method. Plasma AA were measured by ultrahigh performance liquid chromatography (UPLC), while plasma GI hormones were measured using a flow cytometric multiplex array. Following meat ingestion, the circulatory concentrations of some of the essential AA (all the branched-chain AA: leucine, isoleucine and valine; and threonine), some of the nonessential AA (glycine, alanine, tyrosine and proline) and some of the nonproteogenic AA (taurine, citrulline and ornithine) were increased from fasting levels by 120 or 180 min (*p* < 0.05). There were no differences in circulating AA concentrations between cooking methods. Likewise, of the measured GI hormones, glucose-dependent insulinotropic peptide (GIP) and glucagon-like peptide-1 (GLP-1) concentrations increased from fasting levels after consumption of the steak sandwich (*p* < 0.05), with no differences between the cooking methods. In the healthy male adults, protein digestion and circulating GI hormone responses to a beef-steak breakfast were unaltered by the different cooking methods.

## 1. Introduction

Red meat, including beef, is a major source of dietary protein in the western world [1]. The processing and cooking of different cuts of red meat significantly influences the physicochemical properties of the resultant consumed food product [2,3]. The relationship between textural and tenderness changes during cooking is complex, and is regulated by the interactions of heat and time on various components, including connective tissues, myofibrillar proteins and the lipid matrix [2,3,4,5].

High temperature dry cooking causes a rapid protein denaturation, and a loss of the water-holding capacity within the meat. Therefore, in response to pan-frying (PF) the meat is initially softer with cooking (rare) but with a longer cooking time (well-done) it becomes increasingly tough [6,7]. Alternatively, moist-heat cooking techniques (e.g., sous-vide (SV)) are reliant on a lower temperature and longer periods of cooking, which tend to aggregate and gelatinise the sarcoplasmic proteins. With these methods, increasing cooking time increases meat tenderness [6,8]. 

Previous studies have shown that the extent and nature of meat cooking impacts on the measured rate of in vitro protein digestion [4,8]. These studies are supported by preclinical analyses in pigs and rats, with higher-temperature-cooked meat reducing protein digestibility [9,10]. Collectively, these studies suggest that the compositional and microstructural variation in red meat, arising from different cooking methods, will influence digestive responses. 

In addition to the liberation of macronutrients, including protein, red meat is also likely to influence the production of a range of appetite regulatory hormones from the gastrointestinal (GI) tract. These include glucose-dependent insulinotropic peptide (GIP), glucagon-like peptide-1 (GLP-1), peptide tyrosine-tyrosine (PYY), amylin and ghrelin, all of which are involved in aspects of protein-specific appetite regulation [11,12].

The aim of the current study was to investigate whether differences in meat cooking methods could exert an impact on the postprandial plasma concentrations of AA and GI hormones in healthy subjects. Analysis was performed on circulating AA and GI hormones, using samples obtained from a previously reported human crossover trial [13,14] in which healthy young men were randomly assigned to consume 270 g of beef rump steak cooked by two contrasting methods: PF or SV. We hypothesised that the SV-cooked beef steak would be more rapidly digested than the PF beef steak.

## 2. Materials and Methods 

### 2.1. Study Design and Treatments

The clinical study has previously described outcomes of the plasma’s elemental response [14] and inflammatory factors [13]. In this manuscript, we report on the secondary outcome variables of plasma AA and GI hormone responses. Participants were enrolled after a primary screening based on the following exclusion criteria: willingness to eat beef; ability to donate blood; and freedom from any serious medical condition, including cardiovascular disease, diabetes, or obesity. Fourteen healthy young men, aged between 18 and 25 years and with a BMI 18.5—24.9 kg/m^2^, were recruited. The guidelines of the Declaration of Helsinki, and all procedures involving human subjects in the clinical trial, were followed. Ethics approval was obtained from the University of Auckland Human Participants and Ethics Committee (reference number: 7947), and all subjects provided written informed consent. This trial was registered with the Australian New Zealand Clinical Trials Registry (ANZCTR12612000534886), and the study was conducted in 2012. 

**Figure 1:**
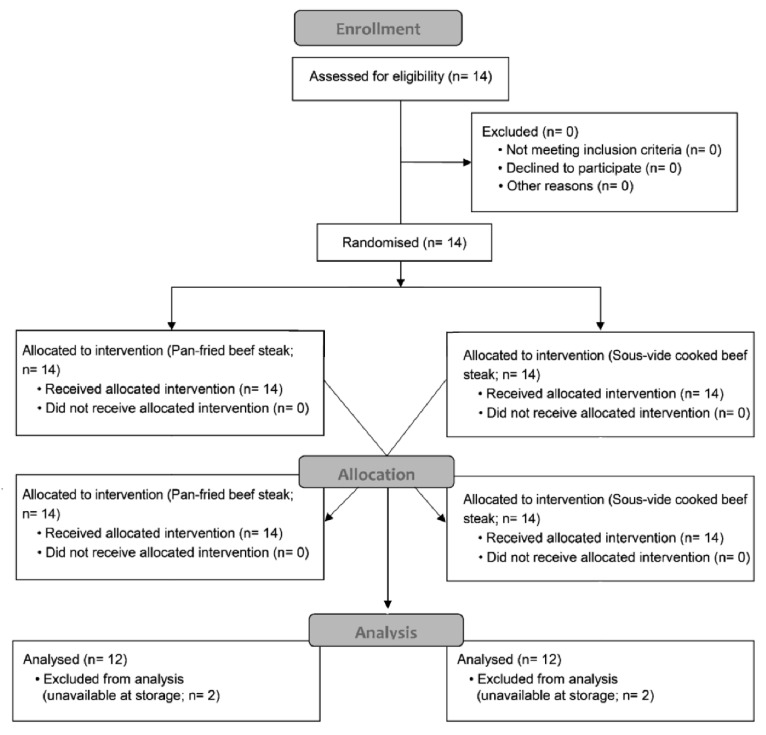
Consolidated Standards of Reporting Trials (CONSORT) diagram of the study participants from enrollment, allocation and sample analysis.

The sequence of the intervention was randomised by a computerised sequence generation (www.randomizer.org), and concealed by use of sealed envelopes. Following enrolment, the subjects were allocated to the intervention meals, with no masking, in a crossover fashion. Participants were asked to maintain a one-week washout period before each clinical visit. On each morning of the clinical visit, participants presented at the Human Nutrition Unit (University of Auckland) after an overnight fast. Pre-intervention (baseline) blood samples were collected from an antecubital arm vein, and a steak sandwich was provided. Postprandial blood samples were then collected hourly for 4 hours after ingesting the meal. The participant enrolment and randomisation, including the number of samples available for this analysis, is presented in Figure 1.

Blood was collected in EDTA tubes (BD Vacutainer®, Becton, Dickinson and Co., Franklin Lakes, NJ, USA), centrifuged at 3000 × *g* for 15 min at 4 °C within 20 min of the blood draw and stored at −20 °C prior to the analysis. 

### 2.2. Meat Procurement and Meal Preparation

Raw beef rump steaks trimmed of the fat border were purchased from a local supplier (AFFCO Meat Processing and Products, Hamilton, New Zealand). This cut is typically very lean with little intramuscular marbling. The beef originated from Angus–Friesian crossbreeds, raised primarily on pasture in the Waikato region of New Zealand. The meat was cooked, either by a PF or SV method. For PF, a nonstick frying pan was preheated to 240 °C. The steak was grilled on one side for 3 min and the second side for a further 2 min. For SV, a vacuum packaged steak was immersed into a preheated water bath maintained at 80 °C and cooked for 6 h. The test meal (a steak sandwich) comprised a beef rump steak (270 ± 20 g pre-cooked weight) presented between two slices of plain white bread (50 g, Homebrand, Progressive Enterprises Limited, Manukau, New Zealand) and served with 20 g of tomato sauce or ketchup (Delmaine, Auckland, New Zealand). The test meal provided 608.4 kcal energy, and protein, fat, and CHO contributed 49.1%, 29.6% and 20.2% of the calories, respectively (FoodWorks v10, Xyris, Australia).

### 2.3. Biochemical Analysis

Plasma-free AA (essential AA [EAA]: leucine, isoleucine, valine, phenylalanine, methionine, lysine, histidine and threonine; nonessential AA [NEAA]: glycine, asparagine, alanine, arginine, serine, proline, tyrosine, aspartic acid, glutamic acid and glutamine; nonproteogenic AA: taurine, hydroxyproline, ornithine and citrulline) were measured by the ultrahigh performance liquid chromatography (UPLC) technique following a standard protocol, as described previously [15,16]. A fluorescence detector coupled with a Kinetex EVO C18 1.7 µm 150 × 2.1 mm separation column (Thermo Scientific Dionex Ultimate 3000 pump; Thermo Fisher Scientific, Dornierstrasse, Germany) was used. Spectra were analysed with Chromeleon 7.1 software (Thermo Fisher Scientific), to quantify the AA concentrations compared to the standard curves created from the mixed standards (Sigma Chemical Company, St. Louis, MO, USA).

Plasma insulin and the GI hormones (GIP: glucose-dependent insulinotropic peptide, GLP-1: glucagon-like peptide-1, PYY: peptide tyrosine-tyrosine, ghrelin and amylin) were analysed simultaneously using a flow cytometric multiplex array (Milliplex^®^ MAP Kit; Human Metabolic Hormone Magnetic Bead Panel Assay; HMHMAG-34K; Millipore, Billerica, MA, USA), with MAGPIX^®^ Luminex multi-analyte profiling (xMAP) technology, following the manufacturer’s instructions. The generated fluorescent intensity data were analysed using Milliplex^TM^ Analyst Software xPONENT^®^ System Version 3.5 (Thermo Fisher Scientific, Waltham, MA, USA), to calculate the analyte concentrations in the samples. 

### 2.4. Statistical Analysis

Based on an 80% power to detect a difference in protein digestion and absorption kinetics, at least 10 subjects would be required with a two-sided significance level of 0.05. The calculation was based on a reported difference in the mixed muscle protein fractional synthetic rate (FSR) of 20% and an SD of 15% [17]. Statistical outliers were identified and removed using three times the interquartile range (IQR), and the linear mixed-effects model analysis was used to account for missing data points. Linear mixed-effect model analyses were performed to compare concentration data variables (time points × cooking methods), followed by Sidak adjusted multiple comparison posthoc tests using SPSS Statistics 25 (IBM Corp., Armonk, New York, NY, USA). When no interaction was observed, there was however a fixed effect of time, and so posthoc tests were performed to identify the time-points at which variable concentrations were significantly different from the baseline. The baseline-adjusted area under the curve (iAUC) was also calculated for the AA, and these data were compared using a paired *t*-test to detect differences between responses. α was set at 0.05, and GraphPad Prism version 7.0 for Windows (Graph Pad Software, San Diego, CA) was used for generating figures. The data were presented as mean ± SEM.

## 3. Results

### 3.1. Plasma Amino Acid Concentrations

**Figure 2:**
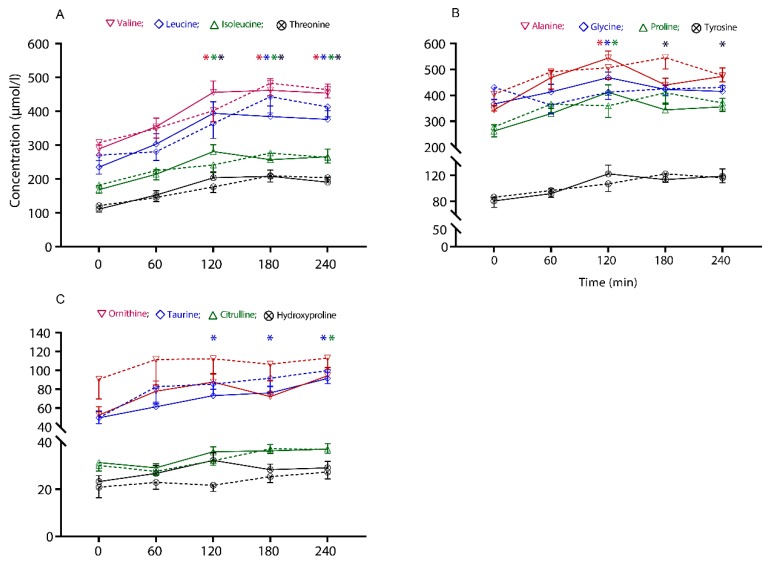
The concentrations of plasma-free amino acids across the intervention period in response to the two treatment arms, SV: sous-vide (solid lines); PF: pan-frying (dashed lines). The values are presented as mean ± SEM concentration (µmol/l) of (**A**) essential amino acids (valine, leucine, isoleucine and threonine), (**B**) nonessential amino acids (alanine, glycine, proline and tyrosine), and (**C**) nonproteogenic amino acids (ornithine, taurine, citrulline and hydroxyproline). Comparisons between the two cooking methods and interactions with time (time × cooking methods) were conducted with linear mixed-effects model analysis. * The times at which the amino acid concentrations significantly differ from the baseline (*p* < 0.05, posthoc is relevant to the main time effect only).

Following the test meal, circulating concentrations of any of the AA did not differ between the cooking methods; as such, no interaction (time × cooking methods) was observed (*p* > 0.05 for all the AA). However, there was a fixed effect of time for an increase in some of the AA concentrations, as shown in Figure 2. There were no differences in the baseline-adjusted area under the curve (iAUC) for any of the AA in response to the different cooking methods, over the 240 min period.

Circulatory concentrations of the EAA, including the branched-chain AA (BCAA: leucine, isoleucine and valine) and threonine, increased with time and were above baseline from 120 to 240 min, in response to both beef steak sandwiches (*p* ≤ 0.006). Other circulatory EAA (phenylalanine, methionine, lysine and histidine) showed a postprandial increase, but not in a manner that differed from their baseline concentration (*p* > 0.05 for all the AA).

In response to consumption of both beef steaks, among the nonessential amino acids (NEAA), alanine, glycine and proline concentrations were above baseline at 120 min, and, tyrosine was above baseline at 180 and 240 min (*p* ≤ 0.047). Other circulatory NEAA (asparagine, arginine, serine, aspartic acid, glutamic acid and glutamine) showed a postprandial rise; however, they did not differ from their baseline concentration (*p* > 0.05 for all the AA). 

Of the nonproteogenic AA, circulatory concentrations of taurine increased above the baseline from 120 to 240 min in response to both test meals (*p* ≤ 0.011), and citrulline concentration increased above the baseline at 240 min (*p* ≤ 0.047), whereas ornithine and hydroxyproline did not differ from their baseline concentration (*p* > 0.05). 

### 3.2. Plasma Gastrointestinal Hormone Concentrations

Plasma insulin concentrations increased rapidly after the meal, peaking within 60 min following the consumption of both SV-cooked and PF beef (*p* ≤ 0.001). Subsequent measurements at 120 and 180 min demonstrated a fall towards the baseline values; however, they differed significantly from the baseline (*p* ≤ 0.001). 

**Figure 3:**
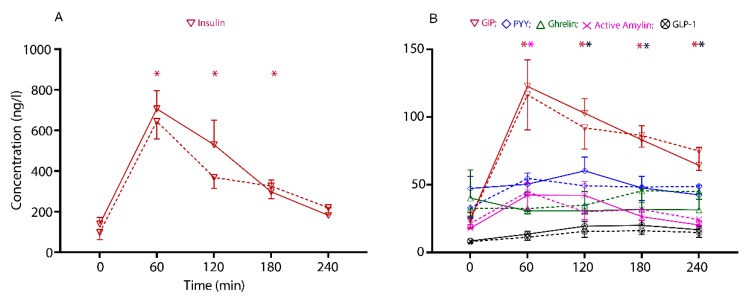
The circulatory concentration of gastrointensinal (GI) hormones across the intervention period in response to the two treatment arms, SV: Sous-vide (solid lines); PF: pan-fried (dashed lines). The values are presented as mean ± SEM of: (**A**) Insulin (ng/L), and (**B**) GIP: gastric inhibitory protein, PYY: peptide tyrosine-tyrosine, ghrelin, active amylin and GLP-1: glucagon-like protein-1 (ng/L). Comparisons between the two cooking methods and interactions with time points (time × cooking methods) were conducted with linear mixed-effects model analysis. * The times at which the hormone concentrations significantly differed from the baseline (*p* < 0.05, posthoc is relevant to the main time effect only).

No postprandial differences were observed in plasma concentrations of the GI hormones (GIP, GLP-1, active ghrelin, PYY and active amylin) between the cooking methods (time × cooking method, *p* > 0.05). However, there was a fixed effect of time for an increase in some of the GI hormone concentrations, as shown in Figure 3. GIP concentrations increased almost five-fold over the baseline at 60 min (*p* = 0.001), and remained different from the baseline until 240 min (*p* ≤ 0.002), for both the SV and PF cooked beef.

**Figure 4:**
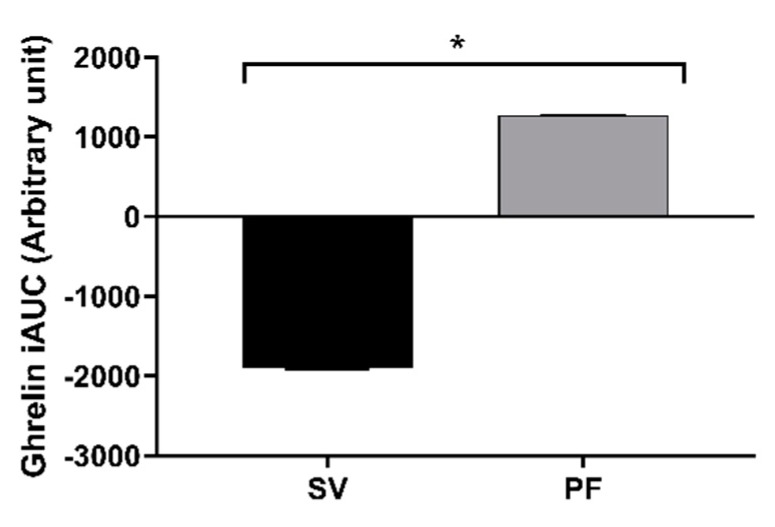
The baseline adjusted area under the curve (iAUC_0-240_) of the circulatory active ghrelin hormone in response to the two treatment arms, PF: pan-fried and SV: sous-vide. The values are presented as mean ± SEM. Comparisons between the two cooking methods were conducted using a paired *t*-test. * The values differed significantly between the cooking methods (*p* = 0.006).

The iAUC of the active ghrelin demonstrated a difference between the cooking methods (*p* = 0.006), and was suppressed with the SV cooked beef steak consumption, as shown in Figure 4, whereas the iAUC of no other GI hormones, including insulin, differed between the cooking methods over the 240 min period.

## 4. Discussion

Meat, as a high-quality protein source, supports muscle protein synthesis and, correspondingly, has the capacity to improve quality of life through improving lean body mass and physical performance [18,19]. However, there has been considerable recent attention on the possible health consequences of a diet rich in red meat, including beef [20,21], yet surprisingly little is known about beef digestion. In this study, a comparison was made of a beef steak prepared using two different cooking methods on the circulatory AA and plasma GI hormones in the four hours following ingestion. We demonstrated that after consumption of a beef steak sandwich (along with two slices of plain white bread), there was a robust increase in the circulating concentrations of insulin and GIP, with modest (less than two-fold) changes in some plasma AA, over the 4 hour analysis period. In contrast to the hypothesis that the cooking methods would influence the digestive and hormonal response, this study measured no significant differences.

The current study measured only the circulatory concentrations of AA, and thus it was not possible to precisely determine the relative contribution of AA appearing from the food or the contribution exerted by changes in the concentration of endogenously derived AA. However, a circulatory upsurge of major EAA within two hours of meat consumption demonstrated the efficacy of both the cooking methods on meat digestibility. Similarly, this current study provided no indication of the total ileal digestibility of each type of cooking method, although it has been previously demonstrated that the ileal digestibility of bovine meat can be moderately decreased by high temperature and long cooking time [22]. It has also previously been shown that the tissue shrinkage from high-temperature meat cooking modifies inter- and intraprotein interactions, potentially inhibiting enzymatic proteolytic cleavage [4]. As previously reported from this study, there was a greater lipid oxidation with high-temperature cooking [13]. It was likely that there was also protein carbonyl compound generation; polymerisation and peptide scissions; and the irreversible modifying of EAAs such as lysine, threonine and arginine [23]. Moreover, increased surface hydrophobicity, carbonylation, protein aggregation and Schiff base formation have also been observed with high-temperature cooking [3,24]. These characteristic protein modifications in cooked meat have been shown to lower protein digestibility in vitro [23,25,26]. Thus, while it is likely that the in vitro digestibility of the high-temperature PF cooked beef steak used in the current study may have been reduced, we were unable to provide any evidence of differences in the measures of total digestibility in vivo.

Furthermore, moist cooking of meat with high connective tissue content was shown to induce a significant tenderising effect via collagen gelation [27], liberating collagen peptides with higher levels of AA (glycine, proline, hydroxyproline and alanine) [28]. In contrast to our hypothesis that protein breakdown would be accelerated with SV, AA indicators of protein breakdown, such as collagen breakdown, including hydroxyproline, did not appear at different rates in circulation. This was surprising given that previous studies had demonstrated that longer periods of cooking solubilise the connective tissues, decrease hardness and make the denatured protein more accessible to digestive enzymes [8,29]. However, in our study, the comparatively lower collagen content of the sampled beef cut [30] prevented our ability to draw firm conclusions. 

This study also explored the dynamics of appetite-regulating GI hormones in response to the different beef cooking methods. Previously, it has been shown that circulatory increases in GIP and GLP-1 (also known as incretins) are proportionate to the caloric content of meals [31,32]. Previous intervention trials have also demonstrated that carbohydrates and fats may be the prominent activators of GLP-1 and GIP secretion [33,34]; however, the role of dietary protein on the secretion of these hormones remains a matter of debate [12,35]. In agreement with previous clinical data [12], our findings show a robust and marked increase in circulatory GIP and a gentle rise in circulatory GLP 1 with the high protein beef steak meals, although there were no differences between the cooking method. Inclusion of white bread, and fat content of the test meal, may also have had an impact on release of GLP-1 and GIP, which needs to be further clarified in future studies.

Unlike other GI hormones, ghrelin concentrations in plasma increase with prolonged fasting and tend to be suppressed by meal intake [36]. There is one report for an increase in ghrelin concentration following a high protein meal [37]. Conversely, other studies have reported a similar response to meals of various macronutrient compositions [38,39]. In this study, the single meal intervention with beef steak did not modify ghrelin relative to fasting concentrations, though it showed a suppression trend throughout the postprandial period. Overall, the present data do not provide evidence for differences in the release of GI hormones following the ingestion of differently cooked beef steak.

### Limitations

This study did not assess the AA composition of the raw and cooked beef steak samples. However, as the meat used was sourced from the same cut of beef, and assigned in a randomised manner, we have assumed that the raw composition was identical. Differences in AA composition as a consequence of cooking methods were also not analysed; thus, the AA composition of the ingested meat could have differed. Further, the GI hormones were analysed from plasma samples immediately after completion of the study, whereas the circulatory AA concentrations were carried out from the prolonged storage of samples at −20 °C, a condition known to influence the concentrations of some AA in plasma [40]. However, all the samples experienced the same storage condition, which likely has minimised the intersample variability.

## 5. Conclusions

Pan-fried and sous-vide beef steak cooking, despite having very different cooking temperatures and times, exerted minimal differential effects on plasma AA, GI hormones, and insulin. This suggests that among healthy young men, variation in meat cooking method is not a major determinant of protein digestion. Previously, in older individuals, preparing beef by mincing was shown to increase the rate of appearance of AA [41]; thus, gross physical changes to meat structure may affect protein digestion to a greater extent than cooking. However, there are important caveats to this study, and further studies are required that combine the use of tracer methodologies or additional measures of total digestibility to more thoroughly examine how meat (including beef) processing and cooking influences postprandial nutrient and hormonal responses.
